# *In vitro* and *in vivo* Pharmacological Activities of 14-*O*-Phenylpropyloxymorphone, a Potent Mixed Mu/Delta/Kappa-Opioid Receptor Agonist With Reduced Constipation in Mice

**DOI:** 10.3389/fphar.2018.01002

**Published:** 2018-08-31

**Authors:** Roberta Lattanzi, Silvia Rief, Helmut Schmidhammer, Lucia Negri, Mariana Spetea

**Affiliations:** ^1^Department of Physiology and Pharmacology “Vittorio Erspamer,” Sapienza University of Rome, Rome, Italy; ^2^Department of Pharmaceutical Chemistry, Institute of Pharmacy and Center for Molecular Biosciences Innsbruck, University of Innsbruck, Innsbruck, Austria

**Keywords:** pain, analgesia, constipation, opioid agonist, opioid receptor, morphinans, binding affinity

## Abstract

Pain, particularly chronic pain, is still an unsolved medical condition. Central goals in pain control are to provide analgesia of adequate efficacy and to reduce complications associated with the currently available drugs. Opioids are the mainstay for the treatment of moderate to severe pain. However, opioid pain medications also cause detrimental side effects, thus highlighting the need of innovative and safer analgesics. Opioids mediate their actions via the activation of opioid receptors, with the mu-opioid receptor as the primary target for analgesia, but also for side effects. One long-standing focus of drug discovery is the pursuit for new opioids exhibiting a favorable dissociation between analgesia and adverse effects. In this study, we describe the *in vitro* and *in vivo* pharmacological profiles of the 14-*O*-phenylpropyl substituted analog of the mu-opioid agonist 14-*O*-methyloxymorphone (14-OMO). The consequence of the substitution of the 14-*O*-methyl in 14-OMO with a 14-*O*-phenylpropyl group on *in vitro* binding and functional activity, and *in vivo* behavioral properties (nociception and gastrointestinal motility) was investigated. In binding studies, 14-*O*-phenylpropyloxymorphone (POMO) displayed very high affinity at mu-, delta-, and kappa-opioid receptors (*K*_i_ values in nM, mu:delta:kappa = 0.073:0.13:0.30) in rodent brain membranes, with complete loss of mu-receptor selectivity compared to 14-OMO. In guinea-pig ileum and mouse vas deferens bioassays, POMO was a highly efficacious and full agonist, being more potent than 14-OMO. In the [^35^S]GTPγS binding assays with membranes from CHO cells expressing human opioid receptors, POMO was a potent mu/delta-receptor full agonist and a kappa-receptor partial agonist. *In vivo*, POMO was highly effective in acute thermal nociception (hot-plate test, AD_50_ = 0.7 nmol/kg) in mice after subcutaneous administration, with over 70- and 9000-fold increased potency than 14-OMO and morphine, respectively. POMO-induced antinociception is mediated through the activation of the mu-opioid receptor, and it does not involve delta- and kappa-opioid receptors. In the charcoal test, POMO produced fourfold less inhibition of the gastrointestinal transit than 14-OMO and morphine. In summary, POMO emerges as a new potent mixed mu/delta/kappa-opioid receptor agonist with reduced liability to cause constipation at antinociceptive doses.

## Introduction

Pain, particularly chronic pain, remains an ongoing global health and socioeconomical problem (Severino et al., 2018), affecting more people than cancer, heart disease, and diabetes combined (Skolnick and Volkow, 2016). Furthermore, comorbidity of chronic pain with mood disorders (e.g., depression, anxiety) in pain patients is well-recognized (Nicholson and Verma, 2004; Tsang et al., 2008; Miller and Cano, 2009). Opioids are the most effective drugs for the treatment of moderate to severe pain (Pasternak, 2014; Stein, 2016). However, their wide use is hampered by unwanted side effects, including constipation, apnea, sedation, nausea, tolerance, and dependence (Benyamin et al., 2008; Imam et al., 2018). A huge increase in medical use and abuse of prescription opioids with raised opioid-related morbidity and mortality has been reported in the past years (Skolnick and Volkow, 2016; Severino et al., 2018). Ongoing monitoring of pain patients receiving opioids to ensure appropriate use and effectiveness is of major importance. The central goal is to balance the patient’s pain relief, potential harmful consequences of opioids, and quality of life. Opioids induce their actions via the activation of opioid receptors, that is, mu (MOR), delta (DOR), and kappa (KOR), as members of the large family of G protein-coupled receptors (GPCRs) with seven transmembrane domains (Kieffer and Evans, 2009; Shang and Filizola, 2015). Opioid receptors modulate neurotransmission in neuronal circuits that subserve pain both at central and peripheral sites (Stein and Machelska, 2011). One long-standing focus of opioid drug discovery is the pursuit for safe and effective analgesics with more favorable pharmacological features. Different approaches are therefore being evaluated to mitigate the deleterious effects of opioid analgesics, with extended reports into the field over the past years (Stein and Machelska, 2011; Aldrich and McLaughlin, 2012; Albert-Vartanian et al., 2016; Del Vecchio et al., 2017; Günther et al., 2017; Madariaga-Mazón et al., 2017; Schmid et al., 2017; Yekkirala et al., 2017; Livingston and Traynor, 2018; Pergolizzi et al., 2018).

**FIGURE 1:**
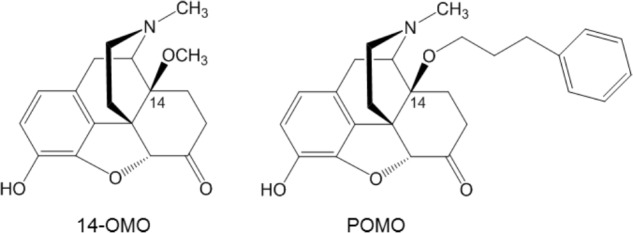
Structures of 14-*O*-methyloxymorphone (14-OMO) and 14-*O*-phenylpropyloxymorphone (POMO).

The MOR is the primary target for analgesia, but also for side effects of opioid analgesics (Pasternak and Pan, 2013). The present understanding of the MOR function is persistently increasing with the crystal (active and inactive) structures of the MOR available (Filizola, 2018). Among clinically used opioids, morphinans including morphine, oxycodone, and oxymorphone, are of key importance as potent MOR agonists (Fürst and Hosztafi, 2008; Spetea et al., 2013). Modifications at position 14 of the morphinan skeleton were targeted by us and others with the prospect of designing novel MOR analgesics, which retain their opioid analgesic properties, but with fewer or no adverse effects (Fürst and Hosztafi, 2008; Lewis and Husbands, 2011; Spetea and Schmidhammer, 2012; Spetea et al., 2013). We have reported that the introduction of a 14-methoxy group in oxymorphone leading to 14-*O*-methyloxymorphone (14-OMO, **Figure 1**) (Schmidhammer et al., 1984) not only increased binding affinity and agonist potency at the MOR, but also resulted in a significant increase in antinociceptive potency in various pain models in rodents (Schmidhammer et al., 1984; Lattanzi et al., 2005; Spetea et al., 2010; Dumitrascuta et al., 2017). However, 14-OMO induces the typical opioid-like side effects (Schmidhammer et al., 1984; Lattanzi et al., 2005). In this study, we describe the *in vitro* and *in vivo* pharmacological profiles of the 14-*O*-phenylpropyl substituted analog of 14-OMO, namely 14-*O*-phenylpropyloxymorphone (POMO, **Figure 1**), which emerges as a new potent mixed mu/delta/kappa-opioid receptor agonist with reduced propensity to cause constipation at antinociceptive doses.

## Materials and Methods

### Drugs and Chemicals

Cell culture media and supplements were obtained from Sigma-Aldrich Chemicals (St. Louis, MO, United States) or Life Technologies (Carlsbad, CA, United States). Radioligands, [^3^H][D-Ala^2^,N-Me-Phe^4^,Gly-ol^5^]enkephalin ([^3^H]DAMGO), [^3^H]5α,7α,8β-(-)N-methyl-N-[7-(1-pyrrolidinyl)-1-oxaspiro(4,5) dec-8-yl]benzeneacetamide ([^3^H]U69,593), and guanosine 5′-O-(3-[^35^S]thio)-triphosphate ([^35^S]GTPγS), were purchased from PerkinElmer (Boston, MA, United States). [^3^H][Ile^5,6^]deltorphin II was obtained from the Institute of Isotopes Co. Ltd. (Budapest, Hungary). Guanosine diphosphate (GPD), GTPγS and opioid ligands, naloxone, DAMGO, [D-Pen^2^,D-Pen^5^]enkephalin (DPDPE), U69,593 and naltrindole, were obtained from Sigma-Aldrich Chemicals (St. Louis, MO, United States). Nor-binaltorphimine (nor-BNI) was purchased from Tocris (Abingdon, United Kingdom). Morphine hydrocloride was obtained from S.A.L.A.R.S. (Como, Italy). Dermorphin and deltorphin I were synthesized as previously described (Erspamer et al., 1989; Negri et al., 1992). 14-OMO and POMO were prepared as described earlier (Schmidhammer et al., 1984; Spetea et al., 2004). All other chemicals were of analytical grade and obtained from standard commercial sources.

### Animals

Male CD-1 mice (20–25 g) and guinea-pigs (400–500 g) were obtained from Charles River (Lecco, Italy, or Sulzfeld, Germany). Animals were housed at 22°C with food and water *ad libitum* and a 12-h light/dark cycle. Animals were used after 4–5 days of acclimatization to the housing conditions. All animal studies were conducted in accordance with ethical guidelines and animal welfare standards according to Italian and Austrian regulations for animal research and were approved by the Animal Care and Use Committee of the Italian Ministry of Health and the Austrian Federal Ministry of Science and Research. All efforts were made to minimize animal suffering and to reduce the number of animals used. For behavioral studies, compounds were dissolved in sterile saline solution, and administered subcutaneously (s.c.) to mice. Separate groups of mice received the respective dose of compound, and individual mice were only used once for behavioral testing.

### Radioligand Binding Assays

Membranes were prepared from Sprague–Dawley rat brains or guinea-pig brains obtained frozen from Labortierkunde und Laborgenetik, Medizinische Universität Wien, Himberg, Austria according to the described procedure (Lattanzi et al., 2005). Protein content of brain homogenates was determined by the method of Bradford using bovine serum albumin as the standard (Bradford, 1976). Binding experiments were performed in 50 mM Tris-HCl buffer (pH 7.4.) in a final volume of 1 ml containing 0.3–0.5 mg protein and various concentrations of test compound as described previously (Lattanzi et al., 2005). Rat brain membranes were incubated either with [^3^H]DAMGO (1 nM, 45 min, 35°C) or [^3^H][Ile^5,6^]deltorphin II (0.5 nM, 45 min, 35°C) for labeling MOR and DOR, respectively. Guinea-pig brain membranes were incubated with [^3^H]U69,593 (1 nM, 30 min, 30°C) for labeling the KOR. Nonspecific binding was determined using 10 μM naloxone. After incubation, reactions were terminated by rapid filtration through Whatman glass fiber filters. Filters were washed three times with 5 ml of ice-cold 50 mM Tris-HCl buffer (pH 7.4) using a Brandel M24R cell harvester (Gaithersburg, MD, United States). Radioactivity retained on the filters was counted by liquid scintillation counting using a Beckman Coulter LS6500 (Beckman Coulter Inc., Fullerton, CA, United States). All experiments were performed in duplicate and repeated at least three times.

### [^35^S]GTPγS Binding Assays

Chinese hamster ovary (CHO) cells stably expressing the human opioid receptors, MOR, DOR, or KOR (CHO-hMOR, CHO-hDOR, and CHO-hKOR cell lines) were kindly provided by Dr. Lawrence Toll (SRI International, Menlo Park, CA, United States). The CHO-hMOR and CHO-hDOR cell lines were maintained in Dulbecco’s Minimal Essential Medium (DMEM)/Ham’s F-12 medium supplemented with fetal bovine serum (FBS, 10%), penicillin/streptomycin (0.1%), L-glutamine (2 mM), and geneticin (400 μg/ml). The CHO-hKOR cell line was maintained in DMEM supplemented with FBS (10%), penicillin/streptomycin (0.1%), L-glutamine (2 mM), and geneticin (400 μg/ml). Cell cultures were maintained at 37°C in 5% CO_2_ humidified air. Binding of [^35^S]GTPγS to membranes from CHO cells stably expressing the human opioid receptors was conducted according to the published procedure (Ben Haddou et al., 2014). Cell membranes were prepared in Buffer A (20 mM HEPES, 10 mM MgCl_2_, and 100 mM NaCl, pH 7.4) as described (Ben Haddou et al., 2014). Cell membranes (5–10 μg) in Buffer A were incubated with 0.05 nM [^35^S]GTPγS, 10 μM GDP, and various concentrations of test compound in a final volume of 1 ml, for 60 min at 25°C. Nonspecific binding was determined using 10 μM GTPγS, and the basal binding was determined in the absence of test compound. Samples are filtered over Whatman glass GF/B fiber filters and counted as described for binding assays. All experiments were performed in duplicate and repeated at least three times.

### Bioassays

Preparations of the myenteric plexus-longitudinal muscle obtained from the small intestine of male guinea-pigs (GPI) and preparations of vasa deferentia of mouse (MVD) were used for field stimulation with bipolar rectangular pulses of supramaximal voltage as described earlier (Lattanzi et al., 2005). Test compounds were evaluated for their ability to inhibit the electrically evoked twitch, and agonist potency was compared with that of the MOR agonist, dermorphin, in GPI, and with the DOR agonist deltorphin I, in MVD. Concentration-response effects were established. All experiments were repeated at least three times.

### Antinociception

Antinociception was assessed using the hot-plate assay performed as described (Lattanzi et al., 2005). Hot-plate latencies were determined by placing each mouse on a hot-plate kept at 55 ± 1°C and observing the occurrence of a nociceptive response (licking of a paw or jumping). Each animal served as its own control. Before drug s.c. administration, each animal was tested, and the basal latency to thermal stimulation was recorded. Animals not responding within 3 s were not used. In order to avoid possible tissue damage, a cut-off time of 12 s was applied. Mice were tested for antinociception after drug administration, and time- and dose-response effect was established. For the antagonism studies, naloxone (1 mg/kg) or naltrindole (3 mg/kg) were s.c. administered 10 min before POMO (2 nmol/kg, s.c.). Nor-BNI (20 mg/kg, s.c.) was administered 24 h before POMO. Antinociception was assessed 20 min after POMO s.c. injection using the hot-plate assay. Doses and pretreatment times of the antagonists were chosen based on pilot studies and previous research (Lattanzi et al., 2002; Erli et al., 2017). Antinociceptive response was expressed as maximum possible effect (%MPE), calculated according to the equation: %MPE = (test latency - basal latency)/(cut-off - basal latency) **×** 100. Each experimental group included six to eight animals.

### Gastrointestinal Transit

The charcoal meal test was used to measure gastrointestinal transit (Broccardo et al., 1998). Mice were fasted for 18 h, with free access to water for the entire study. Animals received 0.25 ml of a suspension of charcoal consisting of 10% (w/v) charcoal suspension in a 5% gum Arabic solution, administered by a gastric tube. Groups of mice were s.c. administered different doses of test drug (morphine: 3900, 6690, and 8000 nmol/kg; 14-OMO: 32, 53, and 90 nmol/kg; POMO: 0.35, 0.70, and 1.6 nmol/kg) or vehicle (saline), 15 min before the charcoal meal, and were sacrificed 15 min later. The stomach and small intestine were separated from the omentum to avoid stretching. The length of the intestine from the pyloric sphincter to the ileocecal junction and the distance traveled by the charcoal meal were measured. The distance traveled by the charcoal meal was expressed as percent of the total length of the small intestine, and the effect was computed as follows: %inhibitory effect = 100 - [(%length traveled after test compound)/(%length traveled after vehicle) × 100]. Each experimental group included eight animals.

### Statistical Analysis

Data were analyzed and graphically processed using the GraphPad Prism 5.0 Software (GraphPad Prism Software Inc., San Diego, CA) and are presented as means ± SEM. For *in vitro* assays, inhibitor constant (*K*_i_ in nM), potency (EC_50_ or IC_50_ in nM), and efficacy (% stimulation) values were determined from concentration-response curves by nonlinear regression analysis. The *K*_i_ values were determined by the method of Cheng and Prusoff (1973). In the [^35^S]GTPγS binding assays, efficacy was determined relative to the reference full opioid agonists, DAMGO (MOR), DPDPE (DOR), and U69,593 (KOR). The AD_50_ defined as the dose that produced an antinociceptive effect equal to 50% MPE in the hot-plate test, the ED_50_ defined as the dose that produced 50% inhibitory effect in the charcol test, and 95% confidence intervals (95% CI) were calculated from dose-response curves (Tallarida and Murray, 1986). Data were statistically evaluated using one-way ANOVA with Tukey’s *post hoc* test for multiple comparisons and unpaired *t*-test for comparisons between two groups, with significance set at *P* < 0.05.

## Results

### *In vitro* Pharmacology – Opioid Receptor Binding and Functional Activity

**FIGURE 2:**
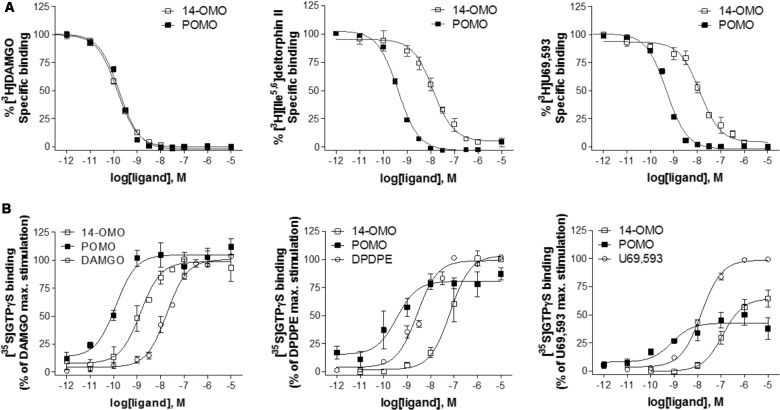
*In vitro* binding and agonist profile of POMO at the opioid receptors. **(A)** Concentration-dependent inhibition by POMO and 14-OMO of [^3^H]DAMGO (MOR) and [^3^H][Ile^5,6^]deltorphin II (DOR) binding using rat brain membranes, and by [^3^H]U69,593 (KOR) using guinea-pig brain membranes, determined in competition binding assays. **(B)** Concentration-dependent stimulation of [^35^S]GTPγS binding by POMO and 14-OMO determined in the [^35^S]GTPγS binding assays using membranes from CHO cells expressing human opioid receptors. Percentage stimulation is presented relative to the maximum simulation of reference agonists DAMGO (MOR), DPDPE (DOR), and U69,593 (KOR). Values are expressed as the mean ± SEM (*n* ≥ 3).

Binding affinity and functional *in vitro* activity of POMO were evaluated at MOR, DOR, and KOR and compared to the profile of 14-OMO. For comparison purposes, the affinity and potency/efficacy data of morphine (Ben Haddou et al., 2014) are also presented. Affinities at opioid receptors were determined in competition binding assays using rat brain (MOR and DOR) and guinea-pig brain (KOR) membrane preparations (Lattanzi et al., 2005). As shown in **Figure 2A**, POMO effectively inhibited in a concentration-dependent manner the binding of selective opioid radioligands to brain membranes. Based on the calculated *K*_i_ values, POMO displayed very high affinity in the picomolar range at the MOR (*K*_i_ = 0.073 nM), similar to the parent compound 14-OMO (*P* > 0.05, *t*-test). However, POMO had also low *K*_i_ values in the subnanomolar range at DOR and KOR, that were significantly lower than the *K*_i_ values of 14-OMO (*P* < 0.05, *t*-test), thus indicating a complete loss of MOR selectivity of POMO when compared to 14-OMO, as well as to morphine (**Table 1**).

**Table 1:** Binding affinities and selectivity of POMO at the opioid receptors.

	Affinity, K_i_ (nM)			Selectivity	
		
	MOR	DOR	KOR	DOR/MOR	KOR/MOR
14-OMO^a^	0.10 ± 0.01	4.80 ± 0.22	10.2 ± 2.0	48	102
POMO	0.073 ± 0.007	0.13 ± 0.02^∗∗∗^	0.30 ± 0.01^∗∗^	1.8	4.1
Morphine^a^	6.55 ± 0.74	217 ± 19	113 ± 9	33	17


*Competition binding assays were performed with membranes from rat brain (MOR and DOR) or guinea-pig brain (KOR). ^*a*^Data from Lattanzi et al. (2005). Values are means ± SEM of at least three experiments. ^∗∗^*P* < 0.01 and ^∗∗∗^*P* < 0.001 vs. 14-OMO (unpaired t-test).*

The opioid agonist *in vitro* activities of POMO were initially assessed on smooth muscle preparations, the GPI and MVD, as well-known widely used bioassays (Leslie, 1987). The GPI is primarily a MOR preparation, even though the ileum also contains KOR. In the MVD, the opioid effects are mostly mediated through the DOR, but MOR and KOR also exist in the tissue. Dermorphin and deltorphin I were used as reference MOR and DOR agonists, respectively. POMO was effective in inhibiting the electrically stimulated twitch in GPI and MVD preparations, with IC_50_ values listed in **Table 2**. In the GPI assay, POMO exhibited potent and full agonist activity at the MOR (IC_50_ = 1.2 nM), with a slight albeit significant increase (*P* < 0.05, *t*-test) than that of 14-OMO. In the MVD preparation, POMO was 1000-fold more potent than 14-OMO as agonist (*P* < 0.05, *t*-test), in line with its enhanced binding affinities at DOR and KOR when compared to 14-OMO. Compared to morphine, POMO was over 250- and 50,000-fold more potent as agonist in the GPI and MVD, respectively (**Table 2**).

**Table 2:** *In vitro* functional activity of POMO at the opioid receptors.

	Bioassay^a^		[^35^S]GTPγS Binding^b^					
		
	IC_50_ (nM)		MOR		DOR		KOR	
				
	GPI	MVD	EC_50_ (nM)	% stim.	EC_50_ (nM)	% stim.	EC_50_ (nM)	% stim.
14-OMO	2.0 ± 0.3^c^	30.5 ± 5.5^c^	1.62 ± 0.48	97 ± 6	43.8 ± 11.7	106 ± 1	144 ± 32	65 ± 7
POMO	1.2 ± 0.21^*^	0.03 ± 0.0013^***^	0.082 ± 0.017^**^	100 ± 8	0.28 ± 0.14^**^	91 ± 8	0.38 ± 0.13^**^	39 ± 5
Morphine	311 ± 29^c^	1600 ± 121^c^	34.4 ± 5.1^d^	89 ± 17^d^	668 ± 65^d^	109 ± 14^d^	710 ± 23^d^	76 ± 2^d^
Dermorphin	1.3 ± 0.27	18 ± 0.31						
Deltorphin I	1239 ± 132	0.19 ± 0.03						
DAMGO			14.7 ± 1.9	100				
DPDPE					1.26 ± 0.76	100		
U69,593							16.7 ± 3.0	100


*^*a*^Functional bioactivity was determined using GPI and MVD preparations. ^*b*^[^35^S]GTPγS binding assays were performed with membranes from CHO stably expressing human opioid receptors. Percentage stimulation (% stim.) is presented relative to the reference full agonists DAMGO (MOR), DPDPE (DOR), or U69,593 (KOR). ^*c*^Data from Lattanzi et al. (2005). ^*d*^Data from Ben Haddou et al. (2014). Values are means ± SEM of at least three experiments. ^∗^*P* < 0.05, ^∗∗^*P* < 0.01, ^∗∗∗^*P* < 0.001, vs. 14-OMO (unpaired t-test).*

In addition to functional bioassays, we assessed the effect of POMO on G protein activation using the ligand-stimulated [^35^S]GTPγS binding assay with membranes from CHO cells stably expressing the human opioid receptors (Ben Haddou et al., 2014). As shown in **Figure 2B**, POMO produced a concentration-dependent increase in the [^35^S]GTPγS binding. Agonist potencies (ED_50_) and efficacies (% stimulation) values are listed in **Table 2**. Stimulation of [^35^S]GTPγS binding was determined and compared to the effect of prototypical full agonists, DAMGO (MOR), DPDPE (DOR), and U69,593 (KOR). POMO was a highly potent agonist at all three receptors, with full efficacy at MOR and DOR, and partial agonism at the KOR (**Figure 2B** and **Table 2**). The *in vitro* functional activity was also affected by the substitution of the 14-*O*-methyl group with a 14-*O*-phenylpropyl group, as POMO showed a significant increase in potency than 14-OMO as defined by the higher EC_50_ values (20-fold at MOR, 151-fold at DOR, and 411-fold at KOR) (*P* < 0.05, *t*-test), while retaining the full agonism at MOR/DOR and partial agonism at the KOR (**Table 2**). Subsequently, the functional MOR selectivity was significantly decreased for POMO.

### *In vivo* Pharmacology – Antinociceptive Activity and Gastrointestinal Transit in Mice

**FIGURE 3:**
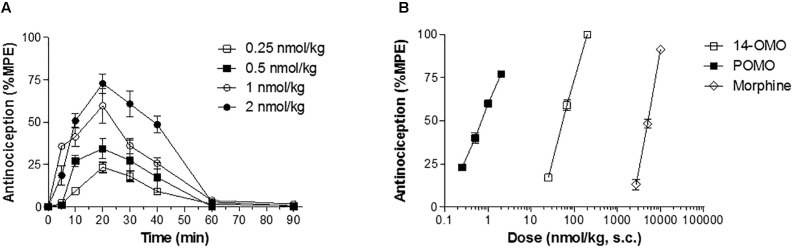
Acute thermal antinociception induced by POMO in the hot-plate assay in mice after s.c. administration. **(A)** Time-dependent antinociceptive effects of POMO. **(B)** Comparison of dose-dependent antinociceptive effects of POMO, 14-OMO, and morphine. Data are shown as mean %MPE ± SEM (*n* = 6–8 mice per group).

POMO was evaluated for antinociceptive activity in a mouse model of acute thermal nociception, the hot-plate assay (Lattanzi et al., 2005). Subcutaneous administration of POMO produced time- and dose-dependent increase in latencies to thermal stimulus, with the peak of antinociceptive response occurring at 20 min (**Figure 3**). Antinociceptive potency as AD_50_ value (and 95% CI) was calculated at the peak of action and compared to 14-OMO and morphine. As shown in **Table 3**, the *in vivo* functional activity was affected by the replacement of the 14-*O*-methyl group with a 14-*O*-phenylpropyl substituent, affording an opioid agonist with more than 70-fold increased antinociceptive potency than 14-OMO. Compared to morphine, POMO was over 9000-fold more effective in producing antinociception in the hot-plate assay in mice.

**Table 3:** Antinociceptive activity and gastrointestinal transit inhibition by POMO in mice after s.c. administration.

	**Antinociceptive activity^a^**	**Gastrointestinal transit^b^**
	
	**AD_50_ (μg/kg, s.c.) (95% CI)**	**ED_50_ (μg/kg, s.c.) (95% CI)**
14-OMO	53 (48–58)^c^	37 (35–39)
POMO	0.70 (0.63–0.77)	1.70 (0.80–3.58)
Morphine	6690 (4468–9348)^c^	3800 (3400–4330)


*^*a*^Determined in the hot-plate assay. Antinociceptive dose 50% with 95% confidence intervals (AD_*50*_, 95% CI), (*n* = 6–8 mice per group). ^*b*^Determined in the charcoal test. Effective dose 50% with 95% confidence intervals (ED_*50*_, 95% CI), (*n* = 8 mice per group). ^*c*^Data from Lattanzi et al. (2005).*

**FIGURE 4:**
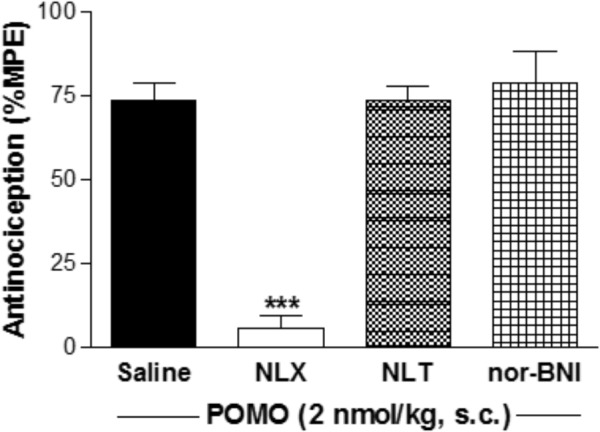
Opioid receptor selectivity of POMO-induced antinociception in the hot-plate assay in mice after s.c. administration. Antinociceptive effect of POMO (2 nmol/kg, s.c.) measured at 20 min was antagonized by pretreatment with naloxone (NLX, 1 mg/kg, s.c., –10 min), but not by naltrindole (NTI, 3 mg/kg, s.c., –10 min) and nor-binaltorphimine (nor-BNI, 20 mg/kg, s.c., –24 h). ^∗∗∗^*P* < 0.001 vs. saline-pretreated group (ANOVA with Tukey’s *post hoc* test). Data are shown as mean %MPE ± SEM (*n* = 6 mice per group).

To determine the relative involvement of the opioid receptor agonist activity in eliciting POMO-induced antinociception, mice were s.c. pretreated with the MOR antagonist naloxone (1 mg/kg), DOR antagonist naltrindole (3 mg/kg), or KOR antagonist nor-BNI (20 mg/kg) prior to POMO s.c. injection, and tested in the hot-plate assay (**Figure 4**). Antinociception induced by the s.c. administration of 2 nmol/kg of POMO was significantly antagonized by naloxone (*P* < 0.05, ANOVA), but not by naltrindole and nor-BNI (*P* > 0.05, ANOVA). Thus, it appears that the activation of the MOR, but not DOR and KOR are responsible for POMO-induced acute thermal antinociception.

**FIGURE 5:**
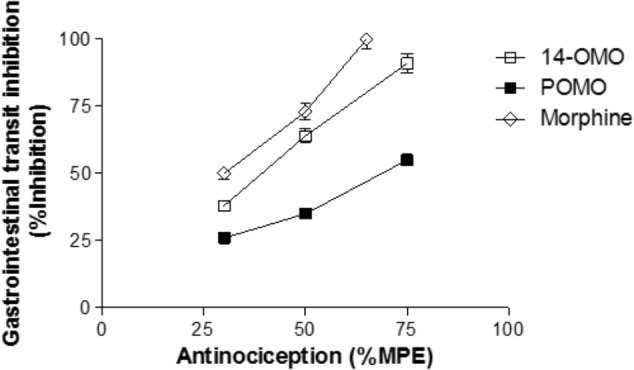
POMO produces antinociception with reduced constipation after s.c. administration in mice. Antinociception (hot-plate assay) vs. gastrointestinal transit inhibition (charcoal test) of POMO, 14-OMO, and morphine. (*n* = 6–8 mice per group).

One of the most frequent adverse effects of opioid analgesics is constipation, as a consequence of activation of opioid receptors in the gastrointestinal tract (Imam et al., 2018). It is well-recognized that the MOR plays a primary role in the inhibitory control of gastrointestinal motility (Imam et al., 2018). *In vivo* studies were performed with POMO by assessing its effect on gastrointestinal transit in mice after s.c. administration using the charcoal test (Broccardo et al., 1998). The inhibitory effective dose, ED_50_ (and 95% C.I.), was calculated and compared to 14-OMO and morphine (**Table 3**). As expected, morphine effectively slowed transit in a dose-dependent manner, with the highest tested dose completely abolishing transit (**Figure 5**). Similarly, and in agreement with our previous data in the colonic bead expulsion test (Lattanzi et al., 2005), 14-OMO dose-dependently inhibited gastrointestinal motility in mice, with the highest dose producing a 90% inhibition. Although POMO also decreases gastrointestinal transit, its actions reached only a 50% inhibition (**Figure 5**).

## Discussion

During the past decades of opioid research, there has been an intensive hunt for an alternative to currently available opioids, which would produce powerful analgesia without the harmful side effects (Bannister et al., 2017; Yekkirala et al., 2017). In this study, we have addressed the exploration of *in vitro* and *in vivo* pharmacological profiles of a new opioid agonist from the class of *N*-methylmorphinan-6-ones, POMO (**Figure 1**). The major finding is that POMO displays potent mixed MOR/DOR/KOR agonism and extraordinarily antinociceptive activity through MOR-mediated mechanisms with considerably reduced propensity for constipation in mice after s.c. administration.

Opioid drug discovery approaches have uncovered that functionalizing position 14 in the morphinan skeleton gives rise to opioid ligands with distinct functional profiles *in vitro* and *in vivo* that are appraised as valuable and potential therapeutics and important research probes (Fürst and Hosztafi, 2008; Lewis and Husbands, 2011; Stavitskaya and Coop, 2011; Spetea and Schmidhammer, 2012; Spetea et al., 2013). Combining *in vitro* ligand binding and functional assays and *in vivo* behavioral approaches, we show that the 14-*O*-phenylpropyl substitution in POMO compared to the 14-*O*-methyl substitution in 14-OMO has a strong influence on the interaction with opioid receptors in terms of receptor binding and activation. The *in vitro* assessment of binding affinities revealed that the introduction of an arylalkoxy group, that is, phenylpropoxy at position 14, maintained the high affinity at the MOR, while markedly increasing affinities at DOR and KOR, hence resulting in a complete loss of MOR selectivity of POMO. These data extend our prior structure-activity relationship (SAR) observations in terms of opioid receptor binding in the series of *N*-methylmorphinan-6-ones when comparing 14-hydroxy and 14-alkoxy analogs (Schmidhammer et al., 1984; Lattanzi et al., 2005; Spetea et al., 2005). Similar to 14-OMO, POMO is characterized as agonist *in vitro* and *in vivo* activity, while exhibiting a distinct functional profile. We showed that *in vitro* functional activity is largely affected by the replacement of the 14-*O*-methyl group with a 14-*O*-phenylpropyl group changing the MOR functionally selective 14-OMO to a potent MOR/DOR full agonist and KOR partial agonist. Notably, POMO exhibited increased affinity and efficacy at the MOR compared to oxymorphone (*K*_i_ = 0.97 nM and EC_50_ = 7.89 nM) (Lattanzi et al., 2005; Dumitrascuta et al., 2017) and morphine (*K*_i_ = 6.55 nM and EC_50_ = 34.4 nM) (Ben Haddou et al., 2014), two clinically used opioids.

Our findings from behavioral studies using a mouse model of acute thermal nociception establish POMO as an extremely potent opioid agonist *in vivo* exhibiting antinociceptive efficacy (AD_50_ = 0.7 nmol/kg) after s.c. administration in mice. Antinociceptive potency of POMO was found to be more than 70-fold higher than that of 14-OMO, and over 9000-fold when compared to morphine. While introduction of a 14-*O*-methyl group in oxymorphone, affording 14-OMO, caused an increase up to 40-fold in antinociceptive potency (Schmidhammer et al., 1984), the presence of the 14-phenylpropoxy group in POMO resulted in a further substantial increase (>1400-fold) than that reported for oxymorphone in the hot-plate assay in mice after s.c. administration (Dumitrascuta et al., 2017). Thus, substitution of the 14-*O*-methyl group in 14-OMO with a 14-*O*-phenylpropyl substituent in POMO leads to a highly potent and efficacious opioid analgesic. The SAR observations derived in this study from the *in vivo* pharmacological findings on antinociceptive properties are in qualitative agreement with the *in vitro* functional activities of targeted opioid agonists. The current findings support and extend our observations on major alterations of the pharmacological profile upon the introduction of a 14-*O*-phenylpropyl group into the opioid antagonists naloxone and naltrexone (Greiner et al., 2003). Hence, naloxone and naltrexone were converted into nonselective ligands with very high affinities at all three opioid receptors, and potent antinociceptive agents in mice after s.c. administration as a result of the presence of the 14-*O*-phenylpropyl substituent (Greiner et al., 2003). However, in the present work, we report on a more thorough evaluation on the consequence of the presence of 14-*O*-phenylpropyl group in *N*-methylmorphinan-6-ones including the mechanism of action for analgesic effects, together with first behavioral studies on the inhibition of gastrointestinal transit. Using pharmacological approaches, we demonstrated that POMO-induced antinociception is mediated through the activation of the MOR, and it does not involve DOR and KOR, as naltrindole and nor-BNI, respectively, did not antagonize the acute thermal antinociceptive effect of POMO in the hot-plate assay in mice.

Prescription opioid use has increased rapidly over the past years (Skolnick and Volkow, 2016; Severino et al., 2018) as have related adverse events including constipation, respiratory depression, tolerance, and dependence (Benyamin et al., 2008; Imam et al., 2018). Respiratory depression is of major concern to clinicians due to its potential for producing fatal outcomes and the primary cause of opioid-related overdose mortality (Imam et al., 2018; Severino et al., 2018). Development of analgesic tolerance pose challenges for compliance and is particularly problematic in long-term chronic pain users (Benyamin et al., 2008; Severino et al., 2018). Opioid-induced constipation is one of the most common and most bothersome side effect of opioid analgesics, and can significantly impact the quality of life (Szigethy et al., 2018). The incidence of constipation is reported in 40–95% of opioid treated patients (Imam et al., 2018). In association with constipation, patients develop other gastrointestinal side effects, including vomiting and nausea, which pose major challenges for compliance and continuation of the therapy for chronic pain management (Imam et al., 2018). All three opioid receptors types, MOR, DOR, and KOR, are present in the gastrointestinal tract of humans (Holzer, 2004; Galligan and Akbarali, 2014). However, opioid-induced inhibition of gastrointestinal transit appears to be mainly mediated by the MOR, as MOR agonists predominantly increase gastric emptying time and inhibit gastrointestinal motility that contributes to nausea and vomiting (Herndon et al., 2002; Imam et al., 2018). In this study, we report on the reduced propensity of POMO to produce constipation at antinociceptive doses after s.c. administration in mice. Based on the calculated ratios of ED_50_(constipation) vs. AD_50_(antinociception) values of 0.54, 0.67, and 2.43 for morphine, 14-OMO and POMO, respectively, it is evident that morphine and 14-OMO cause inhibition of gastrointestinal motility at subanalgesic doses, while POMO showed a larger therapeutic window. Notably, we established that in the charcoal test, POMO produced fourfold less inhibition of the gastrointestinal transit than 14-OMO and morphine in mice.

Evaluation of pharmacokinetics (PK) is an important aspect in drug discovery and development, specially in understanding the behavior of bioactive molecules and correlation with pharmacological activities (Faller, 2008). The *in silico* determination of the partition coefficient (logP) and distribution coefficient at pH 7.4 (logD_7.4_) of 14-OMO and POMO was made using the software MarvinSketch 18.8 (ChemAxon). The calculated logP (*c*logP) values of 14-OMO and POMO were 1.45 and 3.88, respectively, and the calculated logD_7.4_ (*c*logD_7.4_) values were 0.48 and 2.89, respectively, indicative for their good capability to enter the central nervous system. The *c*logP and *c*logD_7.4_ of morphine are 1.23 and -0.57, respectively. Based on the calculated PK parameters, POMO showed a much higher lipophilicity than 14-OMO and morphine, which may account for its pharmacological effects observed *in vivo*.

Herein, we have shown that POMO was highly potent in inducing acute thermal antinociception, via activation of the MOR. *In vitro*, POMO is a mixed MOR/DOR full agonist, as well as a potent KOR partial agonist. The design of ligands that can act at multiple opioid receptors has emerged as a promising new approach to analgesic drug development to potentially lower side effects and to increase analgesic efficacy, especially in chronic pain conditions (Ananthan, 2006; Kleczkowska et al., 2013; Chan et al., 2017; Günther et al., 2017). All opioid receptors, MOR, DOR, and KOR, are crucial modulators of both nociception and opioid analgesia (Pasternak, 2014; Stein, 2016), and are co-localized in nociceptive sensory neurons (Erbs et al., 2015; Massotte, 2015). Compared to the pain relief triggered upon MOR activation in acute pain conditions, agonism at the DOR alone is relatively ineffective (Gavériaux-Ruff and Kieffer, 2011). However, DOR activation can be therapeutically beneficial in the management of persistent inflammatory pain states (Gavériaux-Ruff et al., 2008; Vanderah, 2010), with synergistic agonism at MOR and DOR increasing the overall analgesic effects (Fujita et al., 2015). Activation of the KOR also leads to effective analgesia, especially in visceral pain models (Kivell and Prisinzano, 2010; Yekkirala et al., 2017). Besides, there is less abuse potential, fewer gastrointestinal-related complications and reduced respiratory depression for DOR and KOR agonists compared to MOR agonists (Benyamin et al., 2008; Pasternak and Pan, 2013; Imam et al., 2018). Numerous biochemical and pharmacological studies and studies with genetically modified mice have provided evidence on the modulatory interactions between opioid receptor types, and the existence of MOR/DOR, DOR/KOR, and MOR/KOR heterodimers is recognized (Fujita et al., 2015; Massotte, 2015), and nowadays targeted for the development of bivalent ligands (Ananthan, 2006; Kleczkowska et al., 2013; Fujita et al., 2015; Massotte, 2015; Chan et al., 2017; Günther et al., 2017). On this basis, the activity profile established in this study for POMO as a ligand that can simultaneously bind and activate multiple opioid receptors is of major relevance.

These results provide valuable insights on the SAR in the *N*-methylmorphinan-6-ones class of opioids, by broadening the current understanding of the impact of different substituents at position 14 on ligand-receptor binding, receptor activation and link between antinociception and side effects (i.e., constipation). Future studies remain to analyze in more detail pathway-dependent agonist efficacy and signaling (i.e., biased agonism), effectiveness in models of chronic pain and other opioid typical-side effects. Thus, position 14 in the morphinan scaffold represents a feasible site for tuning functional *in vitro* and *in vivo* activities toward finding effective and safer opioid analgesics.

## Author Contributions

HS, LN, and MS participated in research design. RL, SR, and MS conducted experiments and performed data analysis. HS provided compounds. RL, HS, LN, and MS wrote or contributed to the writing of the manuscript. All authors read and approved the final manuscript.

## Conflict of Interest Statement

The authors declare that the research was conducted in the absence of any commercial or financial relationships that could be construed as a potential conflict of interest.
